# Genomic alterations of primary tumor and blood in invasive ductal carcinoma of breast

**DOI:** 10.1186/1477-7819-8-32

**Published:** 2010-04-21

**Authors:** Ja Seong Bae, Jin Soo Choi, Seung Ho Baik, Woo Chan Park, Byung Joo Song, Jeong Soo Kim, Young Lim, Sang Seol Jung

**Affiliations:** 1 Department of Surgery, The Catholic University, Seoul, Korea; 2 Catholic Neuroscience Center, The Catholic University, Seoul, Korea; 3 Department of Occupational and Environmental Medicine, St. Mary's Hospital, The Catholic University, Seoul, Korea

## Abstract

**Background:**

Genomic alterations are important events in the origin and progression of various cancers, with DNA copy number changes associated with progression and treatment response in cancer. Array CGH is potentially useful in the identification of genomic alterations from primary tumor and blood in breast cancer patients. The aim of our study was to compare differences of DNA copy number changes in blood and tumor tissue in breast cancer.

**Methods:**

DNA copy number changes in blood were compared to those in tumor tissue using array-comparative genomic hybridization in samples obtained from 30 breast cancer patients. The relative degree of chromosomal changes was analyzed using log2 ratios and data was validated by real-time polymerase chain reaction.

**Results:**

Forty-six regions of gains present in more than 30% of the tissues and 70 regions of gains present in more than 30% of blood were identified. The most frequently gained region was chromosome 8q24. In total, agreement of DNA copy numbers between primary tumor and blood was minimal (Kappa = 0.138, p < 0.001).

**Conclusion:**

Although there was only a slight agreement of DNA copy number alterations between the primary tumor and the blood samples, the blood cell copy number variation may have some clinical significance as compared to the primary tumor in IDC breast cancer patients.

## Background

Breast cancer is the most frequently occurring malignancy in Korean women [1]. Even with advances in diagnosis and treatment of breast cancer, the prognosis and survival of patients with breast cancer remains unsatisfactory. Histological and molecular heterogeneity of breast cancer, even in the same stage, hampers the use of standardized treatment. Many women might benefit from more aggressive therapy while others unnecessarily receive treatment. With the aim of individualizing therapy and to refine predictive prognosis, studies have sought to identify biomolecular markers and candidate genes [2-6]. Thus, it is crucial to elucidate the mechanisms involved in breast cancer carcinogenesis at the genetic and molecular levels.

Genomic instability including gain or loss of the region-specific genomic DNA copy number is associated with cancer development and progression [7]. These DNA copy number alterations may result in overexpression of oncogenes with DNA amplification or deletion of tumor suppressor genes [8]. Analysis of DNA copy number changes have been performed using karyotyping, fluorescence in situ hybridization (FISH), comparative genomic hybridization (CGH), and loss of heterozygosity (LOH). However, these methods are limited by their resolution and inability to assess genetic information.

Array-comparative CGH (array-CGH) has been performed to localize DNA copy number changes associated with various human cancers [9-12] and to compare the abundance of specific genomic sequences in whole-tumor DNA relative to normal reference genomes. Array CGH can provide high resolution and dynamic range with more accurate mapping of regions [13-15], and has been used successfully as a tool for the identification of aberrations in breast cancer [16,17].

Array CGH utilizes fresh frozen or formalin-fixed, paraffin-embedded tissue (FFPE) to detect chromosomal alterations in tumor DNA. Although FFPE has been widely used to archive samples obtained from various human cancer, characterization is mainly limited to cytogenetic techniques that analyze genetic changes at the chromosomal level [18,19]. On the other hand, fresh frozen tissues provide the highest quality nucleic acid for analysis. But, clinical availability is often limited. A possible alternative is whole blood samples, since array CGH using whole blood samples has been used for diagnostic testing of patients with mental retardation, birth defects, and behavioral problems [20].

The aim of our study was to compare the chromosomal abnormalities in DNA between fresh frozen tissue and peripheral blood to determine if peripheral blood rather than fresh frozen tissue can be applied for clinical assessment in breast cancer patients.

## Methods

### Sample acquisition

Fresh tissue and peripheral blood samples were each obtained from 30 patients with histologically confirmed breast cancer at the Department of General Surgery at Kangnam St. Mary's Hospital, the Catholic University of Korea following their informed consent. The clinicopathological characteristics of the samples are shown in Table 1. gDNA was extracted from a frozen fragment of the tumor tissue, using a micro-dissection technique to reduce contamination with non-neoplastic tissue. Each tissue sample was incubated overnight at 55°C with cell lysis buffer and 10 μl proteinase K (>600 mAU/ml) (Qiagen, Germany). PBMC was obtained by fycoll hypaque density gradient. Whole genomic DNA was extracted using a Puregene DNA isolation kits (Gentra Systems, USA). The reference sample was used from commercial DNA source (Promega, USA).

**Table 1:** Demographics of patients and tumor characteristics

Characteristic	No. of patients (n = 30)
Mean age (years) ± SD (range)	49.2 ± 8.6 (35--70)
Histological subtype	
Invasive ductal carcinoma	30
Tumor status	
T1	15
T2	13
T3	2
Lymph node status	
N0	17
N1	7
N2	4
N3	2
TNM stage	
I	15
II	9
III	6
Tumor differentiation	
Well	4
Moderate	18
Poor	8
Hormone receptor status	
Estrogen receptor	
Positive	21
Negative	9
Progesterone receptor	
Positive	13
Negative	17
HER-2 receptor status	
Positive	9
Negative	21

### Array CGH analysis

The array used in this study consisted of 4,030 bacterial artificial chromosome (BAC) clones representing duplicates of regions of the whole human genome yielding a resolution of about 1 Mbp. DNA was labeled using the Bioprime labeling kit (Invitrogen, USA). Genomic DNA samples (500~700 ng) with random primers were boiled at 98-100°C for 5 min for denaturation and then cooled on ice for 5 min. The denatured DNA was differentially labeled with 3 μl of 1 mM Cy3 and Cy5 conjugated dCTP (Perkin-Elmer, USA) by random primer labeling, and 1 μl Klenow fragments were added to the mixture. DNA was incubated at 37°C overnight. After labeling, unincorporated nucleotides were removed using MicroSpin G-50 columns (Amersham Biosciences, England). Cy3 and Cy5 labeled test DNA and reference DNA were mixed with 50 μg of human Cot-1 DNA to block repeat sequences. After purification, the mixture was resolved in hybridization buffer containing yeast tRNA to block binding of non-specific nucleotides. MACArray™-Karyo 4 K BAC-chips (Macrogen, Korea) were prehybridized in hybridization buffer with salmon sperm DNA for 30 min prior to hybridization with the purification mixture and incubated for 72 h in a 37°C hybridization chamber. After hybridization was complete, array chips were washed in 50% formamide-2× SSC at 46°C for 15 min, and then 0.1% sodium dodecyl sulfate-2× sodium chloride-sodium citrate (SSC) buffer at 46°C for 30 min. In the next step, the chips were washed in 50% sodium phosphate 0.1% NP40 for 15 min followed by washing in 2× SSC buffer for 5 min at room temperature. After spin drying, hybridized arrays were scanned with a MAC Viewer2™ (Macrogen).

### Data analyses

**Figure 1:**
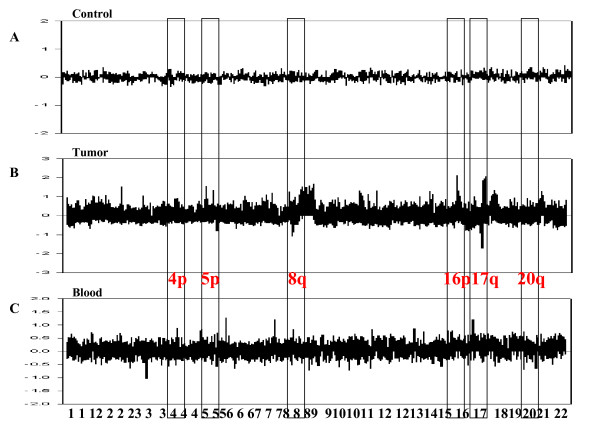
**DNA copy number changes in a representative 30 IDC of each blood and tumor tissue**. Results of array CGH analysis of 30 IDC human breast cancer tumor tissues and blood. The copy number fold change is shown on the y-axis and the genomic location is shown on the x-axis of panels A and B.

The scanned images were analyzed using MAC viewer v.2 Software (Macrogen) to determine the Cy3:Cy5 ratio for each array element. Data were depicted as log_2 _(Cy3 intensity/Cy5 intensity ratios) plotted against the position of clones within the particular chromosome as per the current version of the genome. Based on the ratios of clones mapping to chromosome X in a hybridization of normal female DNA, a specific amplicon was determined (Fig. 1). A ratio of 1.0 indicated a balanced stage of DNA with respect to gain and loss between tissue or blood samples and reference samples; the log_2 _ratio value was plotted as the zero value. A threshold level for determining significant DNA loss was defined as log_2 _ratio < -0.5, while log_2 _ratio > 0.5 represented significant gains. The threshold corresponded to two standard deviations (SD). Centromere regions were excluded from the analysis, which averaged 10 Mb in all chromosomes. The information on each individual clone was obtained from the University of California at Santa Cruz (UCSC) Genome Bioinformatics database http://genome.ucsc.edu.

### Real time-quantitative polymerase chain reaction (PCR)

To confirm genomic imbalances identified by array CGH, DNA samples with obvious genomic changes were analyzed using real time PCR. Primers for clones were selected and the position of each clone was obtained from the UCSC genome database. For relative quantification, the reactions were performed in a total volume of 25 μl that included 12.5 μl of 2 × IQ™SYBR Green^® ^Supermix (Bio-Rad, USA), 1 μl of DNA (10 ng/μl), and 1 μl of each primer (10 pmol/μl). The PCR amplification and detection steps were carried out in an iCycler (Bio-Rad) with 30 s at 95°C, 60°C, and 72°C for 30 cycles after the initial 5 min denaturation step at 94°C. The threshold cycle (C_T_) value was calculated using the comparative C_T _method (Poropat et al. 1998). C_T _for each gene was determined using thermocycler software and an average of three independent experiments was calculated. The N-value of the target gene was normalized to an endogenous reference, glyceraldehyde 3-phosphate dehydrogenase, which shows no significant changes in each genome [21,22].

## Results

Initially, we reviewed the existing literature on oncogenes associated with human breast cancer in breast cancer tissues and compared our results (Table 2). For example, chromosome 8 alterations including a gain of 8q24 are commonly observed in breast cancer [23,24]. The MYC oncogene in the 8q24 region is associated with a worsened prognosis or more aggressive clinical features [25]. This region was amplified in 90% of the presently studied tumors.

**Table 2:** Recurrent gains in breast cancer tissues with examples of some candidate oncogenes.

specimens	Chr.	BAC_Start (bp)	BAC_End (bp)	Size (bp)	Cancer Genes
BC-1;BC-2;BC-5;BC-8;BC-14;BC-17;BC-18; BC-19	1	58958809	59044901	86092	JUN
BC-1;BC-2;BC-3;BC-4;BC-5;BC-6;BC-7;BC-8;BC-9;BC-10;BC-11;BC-13;BC-14;BC-17;BC-18;BC-19;BC-20;BC-21;BC-22;BC-23;BC-25;BC-26;BC-28;BC-30	8	128800101	128877465	77364	MYC
	8	134244673	134344801	100128	WISP1
BC-1;BC-2;BC-5;BC-7;BC-8;BC9;BC-10; BC-17; BC-21;BC-23;BC-25;BC-26;BC-29;BC-30	10	103483027	103545553	62526	FGF8
BC-14;BC-16;BC-17;BC-26;BC-30	11	499204	651925	152721	HRAS
BC-22;BC-26;BC-28	11	69236764	69325605	88841	FGF4
BC-1;BC-10;BC-18;BC-23;BC-29	17	34989583	35061419	71836	STARD3
BC-28	17	31081633	31171045	89412	MMP28
BC-1;BC-4;BC-6;BC-9;BC-10;BC-14;BC19;BC-26;BC-28	17	59297086	59423954	126868	GH1;GH2
BC-26	17	59067300	59150930	83630	MAP3K3

We identified 46 regions of gain present in more than 30% of the primary tumor samples and 70 regions of gain present in more than 30% of blood samples. The most frequently gained region was chromosome 8q24. This region was present in 20/30 (67%) of the tumor samples and 23/30 (77%) of the blood samples. The frequency of copy number loss was lower than that of copy number gains. There were 11 regions of loss found in more than 13% of the primary tumors and 16 regions of loss found in more than 13% of blood (Tables 3 and 4).

**Table 3:** Summary of the most frequent aberrant regions of DNA gain and loss (Blood)

Chromosome	Cytoband	No.of patients (n = 30)	Frequency (%)	Chromosome	Cytoband	No. of patients (n = 30)	Frequency (%)
**Copy number Gain**							
1p	1p36.12	13	13/30 (43)	12q	12q21.2	10	10/30 (33)
	1p36.13	12	12/30 (40)	14q	14q32.12	13	13/30 (43)
	1p36.31	10	10/30 (33)	15q	15q11.2	11	11/30 (37)
	1p35.1	13	13/30 (43)		15q12	10	10/30 (33)
1q	1q23.1	11	11/30 (37)		15q25.2	10	10/30 (33)
2p	2p11.2	11	11/30 (37)	16p	16p11.2	11	11/30 (37)
	2p13.1	14	14/30 (47)		16p13.3	16	16/30 (53)
3p	3p22.1	10	10/30 (33)	16q	16q22.1	12	12/30 (40)
	3p22.2	11	11/30 (37)		16q24.1	13	13/30 (43)
3q	3q21.1	12	12/30 (40)		16q24.3	11	11/30 (37)
	3q21.3	15	15/30 (50)	17p	17p11.2	13	13/30 (43)
	3q27.1	10	10/30 (33)		17p12	11	11/30 (37)
4p	4p16.3	15	15/30 (50)		17p13.2	10	10/30 (33)
5p	5p13.3	12	12/30 (40)		17p13.3	14	14/30 (47)
	5p15.33	17	17/30 (57)	17q	17q11.2	17	17/30 (57)
5q	5q33.1	12	12/30 (40)		17q12	13	13/30 (43)
7q	7q11.23	12	12/30 (40)		17q21.1	10	10/30 (33)
	7q36.3	10	10/30 (33)		17q21.2	11	11/30 (37)
8p	8p21.2	11	11/30 (37)		17q21.32	13	13/30 (43)
8q	8q24.3	20	20/30 (67)		17q25.3	18	18/30 (60)
9p	9p12	11	11/30 (37)	18q	18q23	10	10/30 (33)
9q	9q34.11-9q34.12	17	17/30 (57)	19p	19p13.11	15	15/30 (50)
10p	10p15.3	11	11/30 (37)		19p13.3	16	16/30 (53)
10q	10q22.3	11	11/30 (37)	19q	19q13.2	11	11/30 (37)
	10q26.3	11	11/30 (37)		19q13.33	11	11/30 (37)
11p	11p11.2	12	12/30 (40)		19q13.43	11	11/30 (37)
	11p15.4	12	12/30 (40)	20p	20p12.2	10	10/30 (33)
	11p15.5	18	18/30 (60)	20q	20q13.12	10	10/30 (33)
	11p15.5-11p15.4	12	12/30 (40)		20q13.33	16	16/30 (53)
11q	11q12.3	13	13/30 (43)	22q	22q12.2	19	19/30 (63)
	11q23.1	10	10/30 (33)		22q13.31	15	15/30 (50)
	11q23.3	11	11/30 (37)		22q13.33	15	15/30 (50)
12p	12p13.31	18	18/30 (60)	Xp	Xp11.22	14	14/30 (47)
	12p13.33	12	12/30 (40)		Xp11.23	10	10/30 (33)
12q	12q13.13	10	10/30 (33)	Xq	Xq23	11	11/30 (37)
							
**Copy number Loss**							
1q	1q44	5	5/30 (17)	11q	11q25	6	6/30 (20)
2p	2p25.3	5	5/30 (17)	13q	13q34	6	6/30 (20)
2q	2q37.3	7	7/30 (23)	14q	14q32.33	8	8/30 (27)
3p	3p26.3	5	5/30 (17)	16q	16q21	5	5/30 (17)
5q	5q13.2	6	6/30 (20)	18p	18p11.32	8	8/30 (27)
6p	6p25.3	6	6/30 (20)	21q	21q21.1	5	5/30 (17)
7q	7q22.1	6	6/30 (20)	22q	22q11.1	7	7/30 (23)
10q	10q11.22	8	8/30 (27)		22q11.21	14	14/30 (47)

**Table 4:** Summary of the most frequent aberrant regions of DNA gain and loss (Tissues)

Chromosome	Cytoband	No. of patients (n = 30)	Frequency (%)	Chromosome	Cytoband	No. of patients (n = 30)	Frequency (%)
**Copy number Gain**							
1p	1p36.33	12	12/30 (40)	11p	11p15.5-11p15.4	13	13/30 (43)
1q	1q21.2	12	12/30 (40)	12p	12p13.31	10	10/30 (33)
	1q23.1	14	14/30 (47)		12p13.33	12	12/30 (40)
	1q23.3	12	12/30 (40)	12q	12q21.2	10	10/30 (33)
	1q24.3	12	12/30 (40)	14q	14q32.12	11	11/30 (37)
	1q44	11	11/30 (37)	15q	15q11.2	11	11/30 (37)
2p	2p11.1	13	13/30 (43)		15q12	10	10/30 (33)
	2p25.1	11	11/30 (37)		15q26.3	12	12/30 (40)
3q	3q21.1	12	12/30 (40)	16p	16p13.2	10	10/30 (33)
4p	4p16.3	11	11/30 (37)		16p13.3	23	23/30 (77)
4q	4q32.1	10	10/30 (33)	16q	16q22.1	11	11/30 (37)
	4q35.2	10	10/30 (33)	17p	17p13.3	10	10/30 (33)
5p	5p15.33	24	24/30 (80)	17q	17q11.2	19	19/30 (63)
7p	7p14.1	10	10/30 (33)		17q12	11	11/30 (37)
8q	8q11.1	12	12/30 (40)		17q21.1	11	11/30 (37)
	8q11.21	10	10/30 (33)		17q25.3	18	17/30 (57)
	8q21.3	13	13/30 (43)	18q	18q23	15	15/30 (50)
	8q22.2	17	17/30 (57)	19p	19p13.3	13	13/30 (43)
	8q24.3	23	23/30 (77)	19q	19q13.43	10	10/30 (33)
10p	10p15.3	10	10/30 (33)	20q	20q13.33	21	21/30 (70)
10q	10q26.3	15	15/30 (50)	21q	21q11.2	11	11/30 (37)
11p	11p15.4	10	10/30 (33)	22q	22q13.33	15	15/30 (50)
	11p15.5	14	14/30 (47)	Xp	Xp11.23	10	10/30 (33)
							
**Copy number Loss**							
3p	3p21.31	5	5/30 (17)	16q	16q23.1	4	4/30 (13)
4q	4q35.2	6	6/30 (20)	17p	17p11.2	12	12/30 (40)
6p	6p25.3	5	5/30 (17)	22q	22q11.1	5	5/30 (17)
7q	7q22.1	6	6/30 (20)		22q11.21	11	11/30 (37)
14q	14q32.33	8	8/30 (27)		22q11.23	7	7/30 (23)
16q	16q22.3	4	4/30 (13)				

Thirty other regions of copy number gain were detected in at least 30% of both primary tumors and blood (Table 5). Among these, seven regions of copy number gain were found in more than 50% of both primary tumors and blood. A gain of 5p15.33 was evident in 24/30 (80%) of the primary tumors and 17/30 (57%) blood samples. The region on 5p15.33 was found to contain AHRR, EXOC3 and SLC9A3. A gain at 8q24.3 was frequently detected. This region was found to contain HSF1, DGAT1, SCRT1, FBXL6, GPR172A and ADCK5. A gain of 17q11.2 was evident in 19/30 (63%) of the primary tumors and 17/30 (57%) of the blood samples. The region on 17q11.2 was found to contain MYO18A. A gain of 20q13.33 was detected in 16/30 (53%) of the primary tumors and 19/30 (63%) of the blood samples. This region was found to contain LAMA5, RPS21, CABLES2 and C20orf151. Gain of 22q13.33 was detected in 50% of both primary tumors and blood. This region on 22q13.33 was found to contain MOV10L1, PANX2, TUBGCP6, HDAC10, MAPK12, MAPK11, and PLXNB2.

**Table 5:** Most frequently detected regions in both blood and tissue group by array CGH

Cytoband	Bac_position(Start-End)	Gene	Blood frequency	Tissue frequency
**copy number gain**				
1q23.1	155045002-155148010	SH2D2A, ***NTRK1***, INSRR	11/30(37%)	14/30(47%)
3q21.1	124077821-124170592	***DIRC2***, SEMA5B	12/30(40%)	12/30(40%)
4p16.3	2729092-2810076	SH3BP2	15/30(50%)	10/30(33%)
5p15.33	388661-566921	***AHRR***, EXOC3, SLC9A3	17/30(57%)	24/30(80%)
5p15.33	557250-688780	SLC9A3, CEP72	14/30(47%)	24/30(80%)
8q24.3	145649003-145759358	CYHR1, KIFC2, FOXH1, PPP1R16A, GPT, MFSD3, RECQL4, LRRC14, LRRC24	10/30(33%)	23/30(76%)
8q24.3	145298570-145384455		20/30(67%)	18/30(60%)
8q24.3	145499155-145579895	HSF1, DGAT1, ***SCRT1***, FBXL6, GPR172A, ADCK5	16/30(53%)	19/30(63%)
10q26.3	134654530-134754530	GPR123	11/30(37%)	12/30(40%)
11p15.4	2812494-2941798	KCNQ1, KCNQ1DN, ***CDKN1C***, SLC22A18AS, SLC22A18, PHLDA2, NAP1L4	12/30(40%)	10/30(33%)
11p15.5	499204-651925	***HRAS***, LRRC56, C11orf35, RASSF7, IRF7, MUCDHL, SCT, DRD4, DEAF1	18/30(60%)	11/30(37%)
11p15.5	982365-1053559	AP2A2, ***MUC6***	13/30(43%)	14/30(47%)
11p15.5-11p15.4	2759787-2881783	KCNQ1, KCNQ1DN, CDKN1C, SLC22A18AS, SLC22A18	12/30(40%)	13/30(43%)
12p13.31	6232178-6365032	PLEKHG6, TNFRSF1A, SCNN1A, ***LTBR***	18/30(60%)	10/30(33%)
12p13.33	183679-257363	SLC6A12, SLC6A13	12/30(40%)	12/30(40%)
12q21.2	74611385-74763510	PHLDA1, NAP1L1	10/30(33%)	10/30(33%)
14q32.12	91451809-91569634	FBLN5, TRIP11, PTMAP7	13/30(43%)	11/30(37%)
15q12	24429411-24553848	GABRB3	10/30(33%)	10/30(33%)
16p13.3	979471-1055445		16/30(53%)	23/30(76%)
16p13.3	3369954-3513708	HS3ST4, ZNF434, ZNF174, ZNF597, CLUAP1	12/30(40%)	11/30(37%)
16q22.1	65485602-65560334	CDH16, RRAD, FAM96B, CES2	12/30(40%)	11/30(37%)
17p13.3	907028-1022423	ABR, MRPL14P1	14/30(47%)	10/30(33%)
17q11.2	24429872-24521087	***MYO18A***	17/30(57%)	19/30(63%)
17q21.1	35466169-35565677	***THRA***, NR1D1, CASC3	10/30(33%)	11/30(37%)
17q25.3	78432676-78562724	TBCD, B3GNTL1	11/30(37%)	18/30(60%)
17q25.3	77755881-77849251	SLC16A3, CSNK1D	18/30(60%)	17/30(57%)
19p13.3	5809230-5915258	FUT5, NDUFA11, CAPS, RANBP3	14/30(47%)	13/30(43%)
19q13.43	63514606-63629648	HKR2, A1BG, ZNF497, ***RPS5***, ZNF584	11/30(37%)	10/30(33%)
20q13.33	60334240-60438865	LAMA5, RPS21, CABLES2, C20orf151	16/30(53%)	19/30(63%)
22q13.33	48930979-49068912	MOV10L1, PANX2, TUBGCP6, ***HDAC10***, MAPK12, MAPK11, PLXNB2	15/30(50%)	15/30(50%)
				
**copy number loss**				
6p25.3	202426-307948	DUSP22	6/30(20%)	5/30(17%)
7q22.1	100407386-100480418	MUC12, ***MUC17***	6/30(20%)	6/30(20%)
14q32.33	105821330-105907464	IGHVIII-25-1, IGHV2-26, IGHVIII-26-1, IGHVII-26-2, IGHV7-27, IGHV4-28, IGHVII-28-1, IGHV3-29, IGHV3-30, IGHVII-30-1, IGHV3-30-2, IGHV4-31, IGHVII-31-1, IGHV3-32, IGHV3-33, IGHVII-33-1, IGHV3-33-2, IGHV4-34, IGHV7-34-1	8/30(27%)	8/30(27%)
22q11.1(Cross-Hybridized)	14461738-14573360	DUXAP8	7/30(23%)	5/30(17%)
22q11.21	17158480-17233217	GGT2	14/30(47%)	11/30(37%)

Bold text indicates genes associate with many different carcinoma including breast.

Genomic losses of blood were most often present in 2q (23%), 10q (27%), 14q (27%), 18p (27%) and 22q (47%). Chiefly tumor observed aberration of DNA loss regions in 14q (27%), 17p (40%) and 22q (37%). Only one region of copy number loss was detected in more than 30% of both primary tumors and blood. This region comprised chromosome 22q11.21, and was detected in 11/30 (37%) of primary tumors and 14/30 (47%) of blood. The region on 22q11.21 was found to contain GGT2.

**Figure 2:**
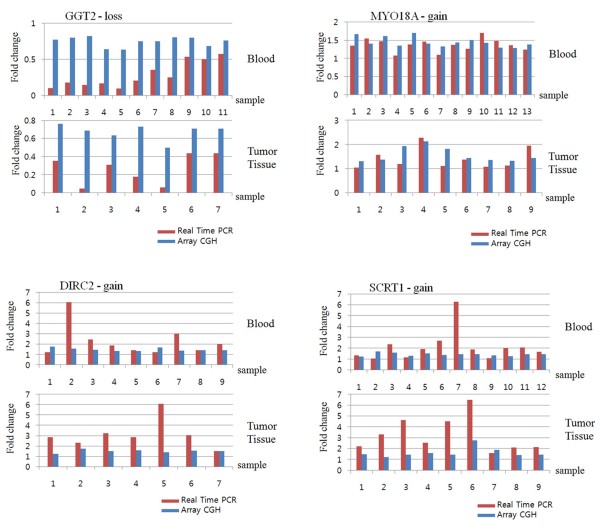
**Comparison of array-CGH with Real-time PCR analysis**. Each sample is depicted on the x-axis, fold change of array CGH is depicted by linear-ratios, and RT-PCR (y-axis) is delineated applying N-value. A threshold level >1 (linear-ration and N-value) indicates significant DNA copy number gain.

To confirm the array CGH results, DNA copy numbers between primary tumor and blood samples were evaluated by real time PCR. As for the array CGH results, several frequently altered loci were found. We selected four related genes that might represent putative candidate genes involved in breast cancer (Fig. 2). Primers for the three genes are presented in Table 6. The clone positions were retrieved from the UCSC genome database (Table 6).

**Table 6:** Primers used for real time PCR analysis

Gene	Forward sequence	Reverse sequence	Region	CNV status
DIRC2	CAGGCAATGGTGAGATCCTG	CCCGAAAACAGGAGGAGAAG	3q21.1	gain
SCRT1	GTGGGGAAGAGGATCAGGAA	CCAGGCTTCAGGGAAGAGAC	8q24.3	gain
MYO18A	GATATCCCCTTGGGCCTGTA	CAGAATGGTGATGCCTCTGG	17q11.2	gain
GGT2	TGGTAGCTTATCCTGGGCCT	ATGGGAGAAGACAGGGATGC	22q11.21	loss

006 March: UCSC genome browser

In the comparison of the results of array CGH with those of real time PCR, the array CGH values were represented by linear-ratios and the N-value was delineated by real time PCR (Fig. 2). The relative fold increases by real time PCR of three genes were consistent with those obtained from array CGH. A parallel examination demonstrated that the gene copy number differences between primary tumor and blood were generally larger when evaluated by real time PCR compared to array CGH. Array CGH and real time PCR corresponded well with respect to chromosomal copy number alterations delineated in each sample.

## Discussion

In this study, we screened for chromosomal aberrations in primary tumors and blood samples obtained from 30 patients diagnosed with breast cancer. Chromosomal abnormalities were evident, with DNA aberrations having a similar tendency to be located at specific chromosomal regions in both sample types.

Genomic DNA copy number changes occur frequently in solid tumors [16] and in association with various human cancers. Recent research has been aimed at determining the phenotype of specific copy number changes [26]. Thus, it has become important to investigate region-specific DNA copy number changes associated with tumor carcinogenesis and prognosis.

Several techniques including FISH, real time PCR, LOH, and CGH have been used to detect DNA copy number changes. Array-CGH is a powerful technique that allows determination of DNA copy number analysis of all regions of large genomes. Unlike conventional CGH, array CGH can provide better resolution and quantitative information at the level of chromosomal gain or loss. Whichever methods are used in the analysis of array CGH data, it is very important that the large volume of data to be validated with various methods including FISH and real time PCR [27,28]. In our study, the latter technique provided the confirmation.

The use of conventional and array CGH for DNA copy number changes in breast cancer is well established, and regions of frequent gain (1q, 8q, 11q, 16p, 17q, 19q and 20q) and loss (6q, 13q, 16q, 17p and 22q) have been identified [13,29-34]. Presently, DNA copy number changes were frequently identified in both primary tumors and blood samples. A large number of regions throughout the genome were altered. DNA copy number alterations of both primary tumor and blood samples were not random. Common DNA gains were more frequently found in 1q, 3q, 4p, 5p, 8q, 10q, 11p, 12p, 12q, 14q, 15q, 16p, 16q, 17p, 17q, 19p, 19q, 20q and 22q, with DNA losses detected in 6p, 7q, 14q and 22q. Seven regions more frequently displayed gain in more than 50% of both the primary tumor and blood samples (Figure 1). Gain on 5p15.33 was identified in 17 cases (57%) of blood samples and 24 primary tumor samples (80%). The region included four genes (AHRR, EXOC3, SLC9A3, and CEP72). AHRR encodes an aryl hydrocarbon receptor repressor, which is a bHLS/Per-ARNT-Sim transcription factor. It was recently reported that AHRR is a putative new tumor suppressor gene in multiple types of human cancers including breast cancer [35].

**Figure 3:**
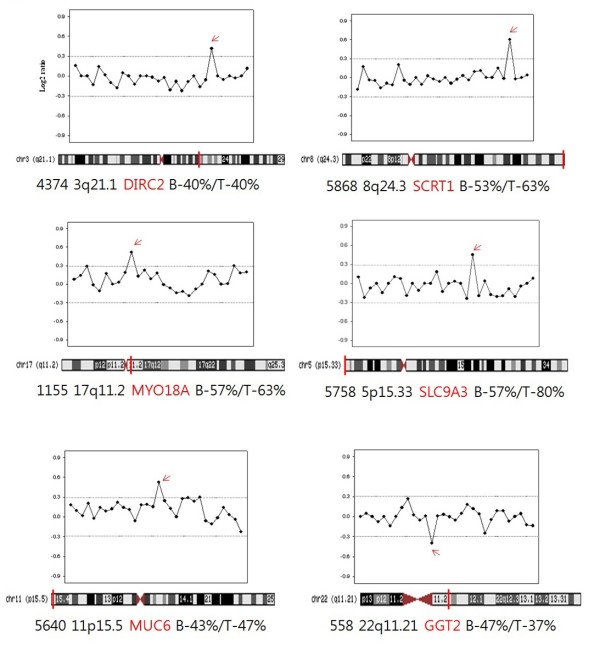
**Frequency of gene copy number abnormalities in some chromosomal regions**. Frequencies of significant genes increased or decreased in copy number at 30 IDC breast cancer samples.

Other candidate genes have been described as frequently imbalanced in the genomes of the breast cancer cells (Figure 3). Gain on 8q24.3 and 20q13.33 was identified in more than 50% of the samples. Gain on 17q11.2 was seen in 19 of the primary tumors (63%) and in 17 of the blood samples (57%). The region included the myosin XVIII A (MYO 18A) gene that is a member of the myosin superfamily, and which has been implicated in atypical myelodysplatic syndrome/proliferative disease [36]. Gain on 17q25.3, which was observed in 17 of the primary tumors (57%) and 18 blood samples (60%), concerns genes encoding solute carrier family 16 member 3 (SLC16A3) and casein kinase 1 delta (CSNGK1D). SLC16A3 is a hypoxia-regulated gene that is expressed in bladder and breast cell lines [37]. CSNK1D is associated with metastasis and relapse of breast cancer, and is overexpressed in lymph node positive breast cancer [38]. Gain on 22q13.33 was over represented in 15 primary tumor and 15 blood samples (50% each). The region includes MOV10L1, PANX2, TUBGCP6, HDAC10, MAPK12, MAPK11, and PLXNB2. Among them, histone deacetylase 10 (HDAC10), a member of class II HDACS, may play a role as a transcriptional modulator in the nucleus and is responsible for lung cancer progression and poor prognosis [39]. In addition, HDACs may also play important roles in cancer development by regulating several genes and causing abnormal gene silencing. A HDAC inhibitor is associated with growth arrest and apoptosis in breast cancer cells [40].

The strength of agreement of DNA copy numbers between primary tumor and blood was slight (Kappa = 0.138, p < 0.001). But we anticipated this result, that the copy number variant size would not be that large nor would it show specific patterns like the private tumor. We have also identified that blood mainly altered regions. Nevertheless, we have also identified the main altered regions in blood samples. 3p22.2 is a region frequently amplified in our blood samples and this region including the MLH1 (mutL homolog 1) is known to be associated with colorectal cancer genes. Gain of 8p21.2 and 9q34.11-12 were also found in blood each about 37% and 57% out of a total of 30 samples (11/30, 17/30). These sites include NKX3-1 (8p21.2) and ABL1 (9q34.11-12) genes that are also known to be associated with prostate tumor suppressor gene and translocation mutation relatively in acute nonlymphocytic leukemia.

Array CGH has been successfully utilized on DNA extracted from fresh-frozen tissues, as these produce high quality nucleic acids [17,34]. However, sometimes fresh-frozen tissues are hard to get and store; more than 70% of the 100-200mg of tissue typically required needs to be comprised with tumor cells. Because the availability of fresh-frozen tissue is limited, the use of FFPE tissue has been explored [19,41]. To date, however, the use of FFPE tissues has been hampered by increased degradation, reduction in the yield of total genomic DNA, and decrease in reliability of DNA [18,42,43].

The results demonstrate the utility of array CGH for detecting DNA copy number changes from primary tumors and peripheral blood, therefore showing the potential use of blood samples in cancer patients in the absence of fresh-frozen tissue. There was a slight agreement of DNA copy number alterations between primary tumor and blood in breast cancer patients. Therefore, further research is necessary for a definitive confirmation of the use of the peripheral blood as a support for primary tumors in identifying putative breast cancer genes through investigation of DNA copy number alterations in a large number of primary tumor and blood samples.

## Competing interests

The authors declare that they have no competing interests.

## Authors' contributions

JSB drafted the manuscript and contributed to conception and design. CJS contributed to acquisition and analysis of data. WCP, BJS, JSK and YL participated in the design of the study and revised ir critically for important intellectual content. SHB participated in the design of study and performed the statistical analysis. SSJ conceived of the study and pariticipated in its design and coordination. All authors read and approved the final manuscript
